# FACTORS RELATED TO THE REDUCTION OF THE RISK OF COMPLICATIONS IN COLORECTAL SURGERY WITHIN PERIOPERATIVE CARE RECOMMENDED BY THE ACERTO PROTOCOL

**DOI:** 10.1590/0102-672020190001e1477

**Published:** 2019-12-20

**Authors:** Alberto BICUDO-SALOMÃO, Rosana de Freitas SALOMÃO, Mariani Parra CUERVA, Michelle Santos MARTINS, Diana Borges DOCK-NASCIMENTO, José Eduardo de AGUILAR-NASCIMENTO

**Affiliations:** 1 Postgraduate Program in Health Sciences, Federal University of Mato Grosso, Cuiabá, MT, Brazil

**Keywords:** Colorectal surgery, Multimodal treatment, Preoperative care, Postoperative care, Postoperative complications, Mortality, Risk factors, Cirurgia colorretal, Tratamento multimodal, Cuidados pré-operatórios, Cuidados pós-operatórios, Complicações pós-operatórias, Mortalidade, Fatores de risco

## Abstract

**Background:**

Perioperative care multimodal protocol significantly improve outcome in surgery.

**Aim:**

To investigate risk factors to various endpoints in patients submitted to elective colorectal operations under the ACERTO protocol.

**Methods:**

Cohort study analyzing through a logistic regression model able to assess independent risk factors for morbidity and mortality, patients submitted to elective open colon and/or rectum resection and primary anastomosis who were either exposed or non-exposed to demographic, clinical, and ACERTO interventions.

**Results:**

Two hundred thirty four patients were analyzed and submitted to 156 (66.7%) rectal and 78 (33.3%) colonic procedures. The length of hospital postoperative stay (LOS) ≥ 7 days was related to rectal surgery and high NNIS risk index; preoperative fasting ≤4 h (OR=0.250; CI95=0.114-0.551) and intravenous volume of crystalloid infused > 30ml/kg/day (OR=0.290; CI95=0.119-0.706). The risk of postoperative site infection (SSI) was approximately four times greater in malnourished; eight in rectal surgery and four in high NNIS index. The duration of preoperative fasting ≤4 h was a protective factor by reducing by 81.3% the risk of surgical site infection (SSI). An increased risk for anastomotic fistula was found in malnutrition, rectal surgery and high NNIS index. Conversely, preoperative fasting ≤4 h (OR=0.11; CI95=0.05-0.25; p<0.0001) decreased the risk of fistula. Factors associated with pneumonia-atelectasis were cancer and rectal surgery, while preoperative fasting ≤ 4 h (OR=0.10; CI95=0.04-0.24; p<0.0001) and intravenous crystalloid ≤ 30 ml/kg/day (OR=0.36; CI95=0.13-0.97, p=0.044) shown to decrease the risk. Mortality was lower with preoperative fasting ≤4 h and intravenous crystalloids infused ≤30 ml/kg/day.

**Conclusion:**

This study allows to conclude that rectal procedures, high NNIS index, preoperative fasting higher than 4 h and intravenous fluids greater than 30 ml/kg/day during the first 48 h after surgery are independent risk factors for: 1) prolonged LOS; 2) surgical site infection and anastomotic fistula associated with malnutrition; 3) postoperative pneumonia-atelectasis; and 4) postoperative mortality.

## RESUMO

**Racional:**

Protocolos multimodais de cuidados perioperatórios melhoram significativamente resultados na cirurgia.

**Objetivo:**

Investigar fatores de risco para vários desfechos clínicos em pacientes submetidos às operações colorretais eletivas com o emprego do protocolo ACERTO.

**Métodos:**

Coorte analisando indivíduos expostos ou não expostos às variáveis de risco demográficas, clínicas e intervenções ACERTO, através de um modelo de regressão logística, determinando fatores independentes de risco para morbidade e mortalidade.

**Resultados:**

Duzentos e trinta e quatro pacientes foram submetidos a 156 (66,7%) operações retais e 78 (33,3%) colônicas. Mantiveram relação com tempo de internação ≥7 dias operação retal e escore NNIS alto; jejum pré-operatório > 4h e volume de cristalóides >30 ml/kg/dia. O risco de infecção de sítio cirúrgico foi aproximadamente quatro vezes maior em desnutridos; oito em operações retais; e quatro com NNIS alto. Tempo de jejum pré-operatório ≤4 h reduziu em 81,3% o risco de infecção de sitio cirúrgico. Risco aumentado para fístula ocorreu em desnutridos, operação retal e escore NNIS elevado. Tempo de jejum pré-operatório ≤4 h constituiu fator de proteção para ocorrência de fístulas. Os fatores associados à pneumonia/atelectasia foram câncer e operação retal, enquanto que tempo de jejum pré-operatório ≤4 h e volume de cristalóides intravenoso ≤30 ml/kg/dia foram fatores de proteção. Mortalidade foi menor com jejum ≤4 h e fluidos endovenosos ≤30 ml/kg/dia.

**Conclusão:**

Este estudo permite concluir que operações retais, presença de fator de risco NNIS, tempo de jejum pré-operatório superior a 4 h e fluidoterapia com cristaloides endovenosos superior a 30 ml/kg/dia nas primeiras 48 h de pós-operatório constituem-se em fatores de risco independentes e aplicáveis para: 1) tempo de internação pós-operatória prolongada; 2) para infecção do sítio cirúrgico e fístula anastomótica associadas à desnutrição; 3) para pneumonia/atelectasia no pós-operatório; e 4) para mortalidade pós-operatória.

## INTRODUCTION

From the last decade on, the use of multimodal protocols of perioperative care have been established as a core part of the routine in various health services in the world^18^. In 2006, Aguilar-Nascimento et al.^2^ reported the first results after implementation of a multimodal protocol of perioperative care conceived in a way to adapt into the epidemiological reality of Latin America. This was named Projeto ACERTO (acronym in portuguese for “Aceleração da Recuperação Total” no Pós-Operatório - Enhancing total postoperative recovery). As ERAS (Enhanced Recovery After Surgery)^25^ ^,^ ^26^ ^,^ ^28^, the ACERTO protocol is based in a solid reference of studies that have shown the benefit of the evidence applied to surgery can positively alter results such as early recovery of organ function, reduced days of hospitalization, decrease postoperative complications and death^3^ ^,^ ^6^ ^,^ ^9^. In a large prospective study with 5,974 elective patients, the same authors compared outcomes before and after the implementation of the ACERTO protocol. The reported a significant improvement of various endpoints such as decrease length of hospital stay (LOS), less need of blood products, and reduced postoperative infections, overall complications and deaths^9^. 

In colorectal surgery these findings are confirmed by various recent controlled randomized trials^17^ ^,^ ^20^ and meta-analyses^19^ ^,^ ^31^ even in studies with the use of videolaparoscopy^21^ ^,^ ^24^. Both in the ACERTO project and ERAS protocol there is a large range of routines in different moments for prescriptions and therefore it is particular interest in investigating which component of one multimodal protocol such as the ACERTO is most associated with the observed beneficial effects. This answer could help for the definitions of better clinical strategies of implementation of programs of enhancing recovery and also, be the basics for decision policies to be adopt aiming at optimization of postoperative recovery^12^ ^,^ ^23^.

Based on these premises, we have proposed to investigate the impact of the main strategies advocated by the ACERTO protocol for postoperative enhanced recovery in the morbimortality after colorectal surgery. We have looked at answering how these strategies related within a multivariate regression model which include clinical and demographic factors that can influence outcome in surgery (age, nutritional status, cancer, etc) as well as including known and recognized prognostic scores that can stratify risk groups. This investigation is particularly interesting because in current literature the studies with the ERAS intervention in colorectal surgery did not reported the impact of prognostic indexes in the analysis of complication rates observed. The same is true for published papers regarding the results of the ACERTO protocol. 

Thus, the aim of this study was to investigate risk factors for various endpoints in patients submitted to elective colorectal procedures within the ACERTO protocol. 

## METHOD

The study was approved by the Mato Grosso Federal University Ethical Committee (protocol number 125646137.000.5541). It is a cohort including patients either exposed or non-exposed to demographic, clinical and ACERTO interventions risk factors through a logistic regression model that assessed independent risk factors for morbidity and mortality. It was included patients submitted to elective open colonic and/or rectal resection followed by primary anastomosis or closure of Hartmann’s colostomy between September 2006 and September 2013. We excluded those underwent palliative procedure without any type of large bowel resection and primary anastomosis, multivisceral resections, abdominoperineal resectium of the rectum, and any surgical treatment of megacolon. Cases with a protective stomas made as an option of the surgical team during either colectomies or rectal resection with primary anastomosis, were also excluded. All subjects were operated on two university hospitals in Cuiabá, MT, Brazil. We selected only cases whose surgeon worked in both hospitals and was actively in charge of surgical procedures and perioperative prescriptions during the period of the study.

. 

### ACERTO protocol

The ACERTO protocol is constituted of various prescriptions including poutines from pre- to postoperative period of surgical patients. In summary the routines are: 1) preoperative fasting time abbreviation (6-8 h for solids and 2 h for clear fluids) - ingestion of a beverage containing 12.5% maltodextrin the night before and again 2 h before anesthetic induction; 2) early postoperative feeding with oral liquid diet in the same day or next day of the procedure; 3) intravenous fluids using Ringer-Lactate solution only until the first postoperative day at a volume of 30 ml/kg/day; 4) no mechanical bowel preparation as routine; 5) nutritional status assessment followed by nutritional therapy for 7-14 days before the procedure for nutritional risk patients; 6) prevention of nauseas and vomiting; 7) antibiotic prophylaxis; 8) deep venous thrombosis prophylaxis; 9) restrict use of drains and nasogastric tubes; 10) early mobilization^2^ ^,^ ^11^. As shown below only some of these routines were studied as risk factors. 

During the study, the adherence to the protocol prescription was at the surgeon discretion in association with the residents in charge with any particular patient. All surgeons were free to manage perioperatice care as confortable as they felt in connection with the multidisciplinar team envolved with the ACERTO protocol in both hospitals. Data were collected in a prospective way using the patients’s files during the years of the study. Cases containing uncompleted, dubious or difficult to understanding data, were excluded. 

### Risk indexes and definitions

The NNIS index (National Nosocomial Infections Surveillance System)^10^, developed by the Center for Diseases Control and Prevention (CDC) was used to assess the risk of infection of the surgical site (SSI). This index ranges from zero to three points based on the presence or non presence three factors. The assessment gives one point if: 1) the procedure is contaminated or infected; 2) the preoperative ASA score (American Society of Anesthesiologists) ranges from 3 to 5; and 3) the duration of the surgery was above the 75 percentile for the estimate procedure time. In this study the cut-off was 3 h. The CR-POSSUM score (Colorectal Physiological and Operative Severity Score for the Enumeration of Mortality and Morbidity)^27^ was assessed to stratify the risk of postoperative morbidity. We used an online calculator found on the website http://www.riskprediction.org.uk to assess this score. 

Surgical site infection (SSI)^5^ ^,^ ^13^ was defined when found until the 30^th^ PO day having: 1) wound purulent discharge with or without laboratorial confirmation; 2) isolation of organism obtained from wound tissue or secretion; 3) at least one of the following parameters incision pain in addition with fever (T >=38^o^ C), edema, hyperemia; 4) purulent discharge in drains; 5) isolation of organism obtained from abdominal cavity tissue or secretion; 6) abscess found during re-operation, histopathological examination or in image scan.

Anastomotic fistula was defined as the presence of abnormal communication between ileo-colic and/or rectal lumen with the exterior through the skin or wound; or intra-abdominal collection containing intestine contents having the anastomosis as the source of the leak and confirmed by laparotomy or any imagen exam. 

Pneumonia/atelectasia was defined as the presence of abnormalities in the lung initiated 48 h after the procedure or 72 h after hospital discharge. These abnormalities had to be seen in radiologic or TC scan and following clinical parameters. Postoperative death was defined as death occurred after the surgical procedure during hospital stay or until 30 days after the surgery since it was related to the procedure. 

### Endpoints

The endpoints of the study were postoperative length of stay (LOS), surgical site infection (SSI), anastomotic leak rate, pneumonia/atelectasia, and postoperative death. LOS was defined was the interval in days between the day of the procedure and day of discharge or death. For statistical purposes LOS was transformed in a categorical variable and analyzed as below or equal the 25 percentile (until 7 days) or above that. 

### Satatistical analysis

For statistical analysis all variables were converted in dichotomic categories (Table 2). For the ACERTO interventions, the dichotomization was based on the median of the preoperative fasting time of 4 h^2^ from the ingestion of the beverage containing 200 ml of 12% maltodextrin until the anesthetic induction. The volume of intravenous crystalloid infusion was divided in ≤30 ml/kg/day or >30 ml/kg/day. Continuous variables were tested for normality by the Kolmogorov-Smirnov (K-S) test and for homogeneity by the Levene test and were expressed in mean and standard deviation (when they were normal and have homoscedasticity) or the median and range (when the distribution was parametric). The categorical data were expressed in number, percent and 95% confidence interval (95CI). All dependent variables were expressed as binary variables (after dichotomization). Firstly, it was assessed the impact of demographic, clinical, and ACERTO intervention factors on the endpoints. The chi-square test or the Fischer’s test when one of the expected values had less then five cases was used to compare the groups. A p<0.05 level was adopt as significant level and the data were expressed as relative risk (RR) and 95% CI. Variables with p<0.25 during univariate comparisons entered a logistic regression analysis (multivariate analysis). This multivariate logistic regression model was done using the mode enter with all covariables reaching p<0,25 at univariate analysis. All covariables were set in the same regression model in one only block and calculation were proceeded accordingly. Residual analysis was done to assess the quality of the model performance on the observed data. The results for each variable elected by each endpoint were expressed in odds-ratio (OR) containing their 95% confidence intervals (95 CI). The Hosmer-Lameshow test was used to assess the adjustment of the data in the regression model comparing expected to observed frequencies. The Nagelkerke (Nagelkerke R^2^) was used to assess the volume of variation explained by the model ranging from 0 to 1. Variables with standard error above 5.0 were excluded of the final analysis to avoid multicollinearity. A value of 5% (p<0.05) was considered as significant in the logistic regression for odds ratios and 95%CI findings. All analyses were done with the SPSS (Statistical Package for Social Sciences) software version 20.0 for Microsoft® Windows^®^. 

## RESULTS

**FIGURE 1:**
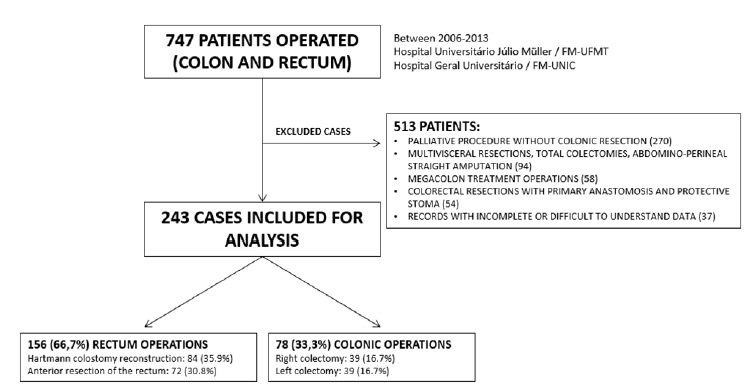
Flow diagram of the cohort study

During the length of the study (2006-2013), 747 patients were submitted to colorectal procedures in the two hospitals. Form these, 513 were excluded due to palliative procedures without colonic resection (n=270), megacolon procedures (n=58), multivisceral resection (including total colectomies) and/or abdominal perineal resection of the rectum (n=58). Another 54 patients were excluded because a protective stoma was built as well as other 37 cases due to errors of the patient’s files (Figure 1, Table 1). A total of 234 patients were then analyzed comprising 84 (35.9%) submitted to Hatmann colostomy reversion, 39 (16.7%) right colectomy, 39 (16.7%) left colectomy, and 72 (30.8%) to anterior resection of the rectum. Therefore, 156 (66.7%) underwent rectal and 78 (33.3%) colonic procedures. 

**TABLE 1:** Demographic, clinical characteristics and ACERTO interventions of the patients included in the study (n=234)

Cases	n=234	
Age (median and variation)	55y	(17-86y)
Gender (n, %)		
Male	157	(32.9%)
Female	77	(67.1%)
Benign diseases	129	(55.1%)
Hartmann colostomy closure after trauma	39 (30.2%)	
Diverticular disease	38 (29.5%)	
Benign tumors	22 (17.1%)	
Others	30 (23.2%)	
Cancer cases (n, %)	105	(44.9%)
Right colon	30 (28.6%)	
Left colon / sigmoid	51 (48.6%)	
Rectal	24 (22.8%)	
Dukes A	9 (8.7%)	
Dukes B	33 (31.4%)	
Dukes C	24 (22.8%)	
Dukes D	39 (37.1%)	
*Subjective Global*Assessment (n, %)		
A	96	(41.0%)
B	87	(37.2%)
C	51	(21.8%)
Colonic operations (n, %)	78	(33.3%)
Right colectomy	39 (50%)	
Left colectomy	39 (50%)	
Rectal operations (n, %)	156	(66.7%)
Anterior resection of the rectum	72 (46.1%)	
Hartman colostomy closure	84 (53.9%)	
NNIS* (n, %)		
0	54	(23.07%)
1	131	(55.98%)
2	39	(16.66%)
3	10	(4.27%)
CR-POSSUM** (median and variation)	1.7480	(0.5000-26.5600)
Preoperative fasting (n, %)		
≤4 h	171	(73.10%)
>4 h	63	(26.90%)
Postoperative feeding (n, %)		
≤24 h	225	(96.20%)
>24 h	9	(3.80%)
Colonic preparation (n, %)	23	(9.80%)
Crystalloid fluids***( mean and standard deviation)	34.65	(±15.43)

*=NNIS index for estimating risk of surgical site infection in colorectal operations; **=CR-POSSUM index to estimate mortality in colorectal operations; ***=volume of crystalloids in ml/kg/day infused in the immediate postoperative and first postoperative day

The median age was 55 (17-86) y/o, 67.1% (n=157) were males and 32.9% (n=77) females. A total of 105 (44.9%) patients had colorectal cancer distribute as follows: 30 (28.6%) in the right colon, 51 (48.6%) in the left colon or sigmoid, and 24 (22.8%) in the rectum. Of those with cancer, nine ((8.7%) were staged as Dukes A, 33 (33.14%) as Dukes B, 24 (22.8%) as Dukes C, and 39 (30.2%) as Dukes D. In the subset of patients bearing benign diseases, 39 (30.2%) underwent Hartmann colostomy closure due to trauma, 22 (17.1%) had diverticular disease, 22 (17.1%) had benign tumors, and 30 (23.2%) had miscellaneous causes. 

The median body mass index was 23 (14-36) kg/m^2^. The nutritional status defined by ASG can be seen in Table 1. A total of 51 (21.8%) of the patients had severe malnutrition (ASG-C). The distribution of the cases according the NNIS infection risk score and the CR-POSSUM mortality index can be seen in Table 2, and also the adherence to ACERTO interventions. There was a high adherence to the abbreviation of preoperative fasting time to 4 h, early postoperative feeding 24 h after the procedure, and reduced postoperative intravenous infusion (<30 ml/kg/day). The mean PO volume of crystalloid intravenous infusion was 34.65 (±15.43) ml/kg/day. However, only approximately 10% of the cases were operated on without mechanical bowel preparation. 

**TABLE 2:** Dichotomized variables for risk analysis of study outcomes, considering demographic, clinical, and interventions.

Variables		n	%	CI95%
Gender	Male	157	32.90	26.9-38.9
	Female	77	67.10	61.1-73.1
Age range	> 60y	85	36.32	30.3-42.7
	≤ 60y	149	63.68	57.3-69.7
Cancer	Yes	105	44.87	38.9-51.3
	No	129	55.13	48.7-61.1
Dukes staging*	A ou B (localized disease)	42	40.00	31.1-49.6
	C ou D (advanced disease)	63	60.00	50.4-68.9
*Subjective Global*Assessment	B ou C (malnourished)	138	58.97	53.0-65.8
(SGA)				
Operation	Rectal	156	66.67	60.7-72.6
	Colonic	78	33.33	27.4-39.3
NNIS score	1.2 ou 3 (higher risk)	180	76.9	71.8-82.1
	0 (lower risk)	54	23.1	17.9-28.2
CR-POSSUM index	>0.9520 (higher risk)	160	68.4	62.4-73.9
	≤0.9520 (lower risk)	74	31.6	26.1-37.6
Preoperative nutrition therapy **	Yes	56	40.6	32.7-48.9
	No	82	59.4	51.1-67.3
Preoperative fasting	≤4 h	171	73.08	21.8-32.1
	>4 h	63	26.92	67.9-78.2
Postoperative feeding	≤24 h (precocious)	225	96.15	1.7-6.4
	>24 h (late)	9	3.85	93.6-98.5
Colonic preparations	Yes	23	9.83	6.4-14.1
	No	211	90.17	85.9-93.6
Crystalloid volume (immediate PO and 1^ST^ PO day)	>30 ml/kg/day	81	34.62	28.6-40.6
	≤30 ml/kg/day	153	65.38	59.4-71.4

*=only cancer patients; **=only malnourished patients (SGA “B” or “C”).

### Endpoints

The median LOS was 9 (interquartile range - IIQ: 7-16) days. In 142 patients (60.7%; CI95 54.3%-66.7%) the LOS was equal or greater than seven days and in 92 (39.3%; CI95 33.3%-45.7%), LOS was lower than seven days. Five cases (2.14%) were re-admitted after 30 days of hospital discharge. 

The number of cases with ISS was 73 (31.20%; CI95% 25.6-37.4). Rate of anastomotic fistula was 25.6% (n=60; CI95 20.5%- 31.6%); 21.8% in rectal and 3.8% in ileal/colonic anastomosis. Sixty-three patients (26.9%; CI95 21.6%-32.9%) had pneumonia/atelectasia and the overall mortality was 6.4% (n=15; CI95 3.9%- 10.3%). 

### Univariate analysis

Table 2 shows the dichotomic variables (demographic, clinical and ACERTO interventions) after univariate analysis to assess risk factors related to the endpoints. 

### Length of hospital stay

**FIGURE 2:**
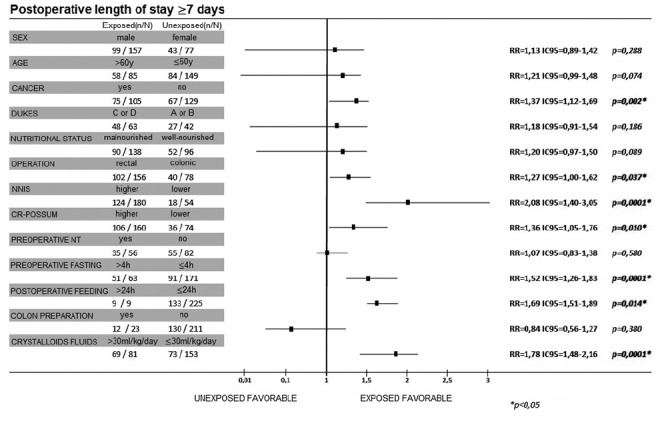
Univariate analysis of risk factors related to postoperative length of stay greater than or equal to seven days

As can be seen in Figure 2, LOS ≥7 days was associated with cancer (RR=1.37; IC95=1.12-1.69; p=0.002), rectal procedure (RR=1.27; IC95=1.01-1.62; p=0,037), NNIS risk (RR=2.08; CI95=1.40-3.05; p=0.0001), high CR-POSSUM index (RR=1.36; CI95=1.05-1.76; p=0.010) and preoperative fasting time greater than 4 h (RR=1.52; CI95=1.26-1.83; p=0.0001). In addition, all individuals fed after 24 h of the procedure had LOS ≥7 days (RR=1.69; CI95=1.51-1.89; p=0.014). In 85.2% (n=69) of the patients the volume of intravenous crystalloids was superior to 30 ml/kg and they had 1.78 times risk of prolonged LOS (CI95=1.48-2.16; p=0.0001). 

### Morbidity

**FIGURE 3:**
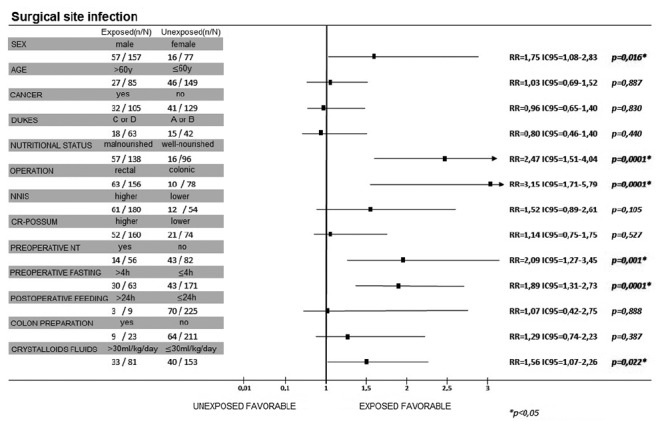
Univariate analysis of risk factors related to surgical site infection

As can be seen in Figure 3, males, malnutrition, rectal procedure, and intravenous crystalloid infusion greater than 30 ml/kg/day constitute risk factors for SSI. Moreover, preoperative fasting time greater than 4 h was associated to a risk 1.89 times greater for SSI (CI95=1.31-2.73; p=0.0001). 

**FIGURE 4:**
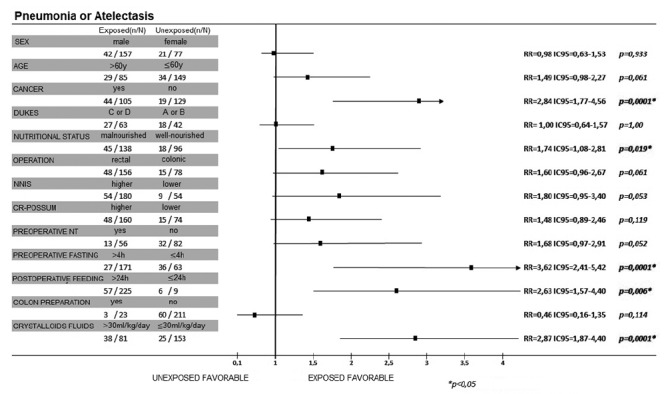
Univariate analysis of postoperative pneumonia / atelectasis risk factors

Preoperative fasting time greater than 4 h (RR=2.71; CI95=1.79-4.11), malnutrition (RR=2.09; CI95=1.24-3.52; p=0.003), and rectal surgery (RR=2.83; CI95=1.47-5.45) were risk factors for postoperative anastomotic fistula. Patients with cancer had 2.84 more risk to have postoperative pneumonia or atelectasia (Figure 4). This risk was 1.74-fold greater in malnourished (CI95=1.08-2.81) and 2.87-fold greater in patients with overload intravenous fluids (CI95=1.87-4.40). As for the ACERTO interventions, the risk of pneumonia/atelectasia was 3.62-fold greater when preoperative fasting was above 4 h (CI95=2.41-5.42; p=0.0001) and 2.63-fold greater in patients fed after 24 h of the procedure (CI95=1.57-4.40; p=0.006, Figure 3).

### Mortality

**FIGURE 5:**
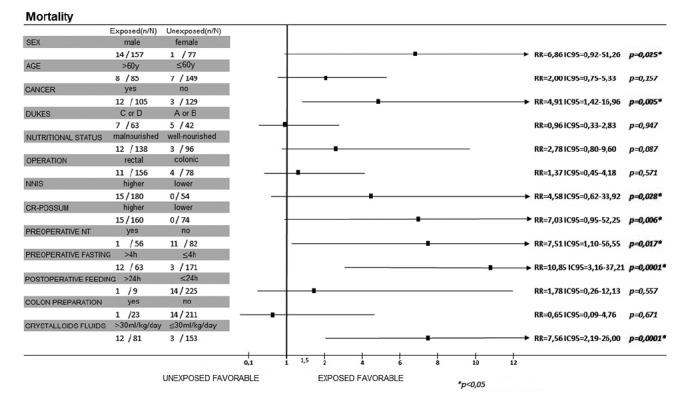
Univariate analysis of mortality-related risk factors

As shown in Figure 5, males (RR=6.86; CI95=0.92-51.26; p=0.025), cancer (RR=4.91; CI95=1.42-16.96; p=0.005), NNIS risk (RR=4.58 CI95=0.62-33.92; p=0.028), and high CR- POSSUM index (RR=7.03 CI95=0.95-52.25; p=0.006) increased the risk of postoperative death. As for the ACERTO interventions, intravenous overload of fluids increased by 7.56-fold the risk of death as well as preoperative fasting time above 4 h (RR=10.85 CI95=3.16-37.21).

### Multivariate logistic regression 

After multivariate regression, rectal procedure (OR=2.93 CI95=1.43-6.02; p=0.03) and NNIS risk (OR=5.25 CI95=2.15-12.86; p<0.0001) have maintained associated to LOS ≥7 days. Preoperative fasting ≤4 h (OR=0.250 CI95=0.114-0.551; p=0.001) and PO crystalloid volume ≤ 30 ml/kg/day (OR=0.290; CI95=0.119-0.706; p=0.006) constituted protective factors for LOS ≥7 days.

The SSI risk was 4.03-fold greater in malnourished (OR=4.03 CI95=1.98-8.20); 8.5-fold greater in rectal procedures (CI95=3.42-21.08), and 4.58-fold greater in patients with NNIS risk (CI95=1.75- 11.97). For ACERTO variables, preoperative fasting ≤ 4 h was a protective factor reducing by 81.3% the risk of SSI in comparison to fasting time above 4 h. The accuracy of the model to predict SSI was 79.1% (61.6% sensitivity and 87% specificity) 

Malnutrition (OR=2.87 CI95=1.36-6.05; p=0.006), rectal surgery (OR=8.23 CI95=3.12- 21.74; p<0.0001), and NNIS risk (OR=6.14 CI 2.09-18.05; p=0.001) increased the risk for anastomotic fistula. Conversely, preoperative fasting ≤ 4 h (OR=0.11 CI95=0.05- 0.25; p<0.0001) in this model was a protective factor for fistula. The accuracy of the model was 79.1% to predict anastomotic fistula. 

Risk factors associated to pneumonia/atelectasis were (OR=4,82 OR=2,03-11,47; p<0,0001) cancer and rectal surgery (OR=3,07 IC95=1,18-7,74; p=0,022). Conversely, preoperative fasting ≤ 4 h (OR=0.10 IC95=0,04- 0.24; p<0.0001) and intravenous volume ≤30 ml/kg/day (OR=0.36 IC95=0.13-0.97, p=0.044) were protective factors for lung complications. 

To preserve the model from multicollinearity the variables NNIS risk and CR-POSSUM index were excluded due to a pattern error above 5.0. The accuracy of the model to predict death was 96.2%; sensitivity of 53.3% and specificity of 99.1%. Cancer had a risk 9-fold greater for PO death than benign conditions (OR=9.04 IC95=1.60-50.89). Other protective factors for PO death in this study were preoperative fasting ≤4 h (OR=0.05 IC95= 0.01-0.23) and intravenous volume ≤30 ml/kg/day (OR=0.14 IC95=0.02-0.97)

## DISCUSSION

The overall findings of this study showed that the endpoints were related to more or less adherence to the ACERTO protocol. Preoperative fasting less ore qual to 4 h was associated with reduced LOS, SSI, anastomotic fistula, and pulmonary complications. The use of a beverage containing maltodextrin was also related with reduced PO death. In addition, intravenous crystalloid fluids infusion less than 30 ml/kg/day was associated with the reduction of LOS by 71% and also was protective to the develop of PO lung complications. Although other ACERTO interventions showed positive effects in the observed endpoints even when adjusted for confounded variables, only these two interventions have maintained the protective effect. 

As expected, other known risk factors also have related with the studied endpoints. We found that NNIS risk and rectal procedure over colonic procedure were associated to prolonged LOS; malnutrition, NNIS risk and rectal procedure were related to SSI and anastomotic fistula; cancer and rectal procedure to pneumonia/atelectasis; and finally cancer over benign conditions was associated to PO death. 

This study comprises patients operated on two hospitals following the first years after the implementation of the ACERTO multimodal protocol. It should be considered that the adherence to the protocol by the surgeons of both services was free which may explain the different percent of the prescribed routines. 

The overall findings showed that a median of nine days for LOS which is superior when comparing to other multimodal protocols^19^ ^,^ ^29^. Nevertheless, this figure is similar to other cohort studies reported in patients undergoing colorectal surgery and under the use of a multimodal protocol^8^ ^,^ ^15^, especially in university hospitals^1^.

The same can be said about the complications rate. Gustafsson et al.^15^ in a prospective cohort study including 953 patients submitted to elective colorectal cancer surgery with the ERAS protocol, reported about 25% of infectious complications and an incidence of 18% of rectal and 3% of colonic anastomotic fistula. 

We have not found studies reporting the effects of a multimodal protocol on the incidence of postoperative pneumonia and/or atelectasis after colorectal surgery In a study published in 2011 which reported the American College of Surgeons NSQIP file on 48.247 patients undergoing open or laparoscopic colectomies, the incidence of pneumonia was 5%^22^. The findings of a higher percent of cases of lung complications in this study may be explained by the definition and sum of cases of postoperative pneumonia plus atelectasis all together. The mortality of the current study was 6.4% (CI 95 3,9%-10,3%). Gatt et al.^14^ reported 5.2% mortality rate in a randomized controlled trial envolving patients submitted to major colonic resections with a multimodal protocol. Another study reported similar mortality rate^16^.

Prolonged LOS was associated with the site of resection (rectal or colonic) and the presence of NNIS risk factor. In fact, colonic or rectal resection have different outcomes and rectal procedures are most prone to increased and more serious over colonic resections^28^. Our findings reinforce this evidence. In this series, not only rectal procedures have been associated to increased LOS but they were an independent factor for SSI, anastomotic fistula, and postoperative pneumonia/atelectasis. NNIS risk index was also an important predictor of complications. This was especially true for infectious complications as having a NNIS risk factor increased by five-fold the risk of SSI. This narrow connection between fistula or postoperative infection with colorectal procedures may be related the finding of at least one NNIS factor present. Nutritional status and cancer are well known factor that can affect surgical outcomes. In Brazil, the incidence of malnutrition in cancer patients can reach 47.6%^30^. In this study, multivariate analysis has shown that malnutrition had a risk four-fold greater for SSI and three-fold greater for postoperative pneumonia/atelectasis. We have also found an increased risk for postoperative pneumonia/atelectasis in cancer patients. 

The benefits of the multimodal approach for the surgical patients seem to be greater when all different elements of the routines are prescribed together as a protocol^2^ ^,^ ^9^ ^,^ ^18^. In a recent meta-analysis, the application of the ERAS protocol in major operations was able to reduce the LOS by 2-3 days, and decrease complications by 30 to 50%^30^. The routines of the ACERTO protocol have shown in earlier studies to significantly decrease LOS, and both infectious and non-infectious complications, and this effect was most expressive in major operations when the entire protocol was executed^2^ ^,^ ^3^ ^,^ ^9^. However, a few studies, in special those in colorectal operations, have evaluated the impact of each one of the prescriptions of the multimodal protocol on the main postoperative endpoints. In this context, Aarts et al.^1^, have evaluated the impact of the ERAS strategies on the LOS in patients submitted to surgery in university hospitals. They observed that the use of videolaparoscopy, preoperative information, intravenous fluid restriction, and the use of oral clear fluids in the day of the surgery were related to a reduction of LOS to five or less days. In this study the chance of early discharge was 1.26 times greater in patients who received less intravenous fluids and 1.09 times in patients who drunk clear fluid on the day of the surgical procedure. 

In a similar way, our findings showed that the infusion of intravenous fluids in a volume ≤30 ml/kg/day during the first 48 h of the procedure and drinking clear fluids 4 h before anesthesia resulted in a reduction of the risk of stay ≥7 days in-hospital by respectively 71% and 75% of cases. 

In agreement, Gustafsson et al.^15^ reported that restriction of intravenous fluids and preoperative fasting of 2 h were the most independent predictors of outcomes after surgery. 

In Brazil, Aguilar-Nascimento et al.^4^, in a multicentric study that included 16 hospitals and 3.715 patients observed that the median preoperative fasting time was 12 (2-216) h. Almost 80% (n=2.962) of the patients were operated on after 8 h of fasting and 46.2% (n=1.718) after 12 h. One can infer that those patients have received an additional volume of intravenous crystalloid fluids of about 25 ml/kg in the day of the surgery. Additionally, prolonged preoperative fasting contributes to increase the chance of postoperative nausea and vomiting^7^, insulin resistance, and negative nitrogen balance and as a result all this can importantly deteriorate the perioperative metabolic stress^11^. 

Our findings support the importance of the abbreviation of preoperative fasting and the correct management of intravenous fluids to decrease postoperative complications and even deaths. Prolonged preoperative fasting (greater than 4 h) was associated to 5.35-fold higher risk for SSI; 9.27-fold for anastomotic fistula; 10-fold for pneumonia-atelectasis; and 20-fold greater risk of postoperative death (OR=20.61; IC95=4.30-98.62). In the same way, intravenous crystalloid fluids infusion greater than 30 ml/kg/day increased by 2.8 times the chance of pneumonia/atelectasis and by seven times the chance of postoperative death (OR=7.12; IC95=1.06-47.96). 

However, the study has limitations due to its long duration (2006 to 2013) because during these years, changes may happened in the two hospital (adherence, changes in staff, etc) and could influenced the outcomes. Moreover, some data were obtained by review of patient’s files and therefore with methodological fragility which can happen in historical cohort studies. For instances, 40 cases were lost due to incomplete information in the files. Finally, a common problem with observational studies of this kind is that of separating the effects of major exposure from those produced by other extrinsic factors or variables. Although the adjustment for several known variables as confounding factors was performed, other extrinsic variables, possibly not studied here, may have masked a possible association between exposure factor and outcomes, over or underestimating the results. 

However, we have not found in literature one study like this which involved a cohort of patients undergoing major elective colorectal surgery, in the context of the use of a multimodal protocol, and considered in the analysis of risk factors, epidemiological (gender, age) and clinical variables (nutritional status, cancer diagnosis, etc.) and in addition, considered also the effect determined by prognostic indexes used to predict severe complications in surgery (NNIS and CR-POSSUM).

In fact, the presence of at least one NNIS risk factor was associated with 5.25 times higher LOS (OR=5.25; CI95=2.15- 12.86); 4.58 times greater SSI (OR=4.58; CI95=1.75-11.97), and 6.14 times greater incidence of anastomotic fistula (OR=6.14; CI95=2.09-18.05). Although the CR-POSSUM index have been associated to prolonged LOS, pneumonia/atelectasis, and postoperative deaths by the univariate analysis, this association was not maintained after multivariate analysis. In this case, we must consider that the original ordinal variable in this study was converted to a nominal variable, through a method that took into account values ​​above or below the first quartile (Q1). Although this is an appropriate method for this purpose, it may have considered patients with low morbidity and mortality probability (lower than the median of the series) as high risk, reducing the accuracy of the prediction method.

Further studies using this tool are needed, assessing its applicability in the context of complication risk analysis, using other cutoffs in patients undergoing multimodal protocols. In addition, the analysis of a more homogeneous population (for example, only cancer patients, or only nutritional deficits), would allow the assessment of multimodal prescriptions taking into account more specific risk factors such as tumor staging and even the performance or not of perioperative nutritional therapy, which could not be done in the present investigation due to the heterogeneity of sampling.

## CONCLUSION

This study allow us to conclude that rectal operations, presence of NNIS risk factor, preoperative fasting time greater than 4 h and fluid therapy with intravenous crystalloids greater than 30 ml/kg/day in the first 48 h postoperatively constitute independent and applicable risk factors for: 1) prolonged postoperative length of stay; 2) surgical site infection and anastomotic fistula associated with malnutrition; c) postoperative pneumonia/atelectasis; and d) and death in colorectal cancer surgery.

## References

[1] Aarts MA, Okrainec A, Glicksman A, Pearsall E, Victor JC, McLeod RS (2012). Adoption of enhanced recovery after surgery (ERAS) strategies for colorectal surgery at academic teaching hospitals and impact on total length of hospital stay. SurgEndosc.

[2] Aguilar-Nascimento de JE, Bicudo-Salomão A, Caporossi C, Silva RM, Cardoso E A, Santos TP (2006). Acerto pós-operatório avaliação dos resultados da implantação de um protocolo multidisciplinar de cuidados peri-operatórios em cirurgia geral. Rev Col Bras Cir.

[3] Aguilar-Nascimento de JE, Bicudo-Salomão A, Caporossi C, Silva RM, Cardoso E A, Santos TP (2008). Enhancing surgical recovery in Central-West Brazil The. ACERTO protocol results.e-SPEN;.

[4] Aguilar-Nascimento JE, de Almeida Dias AL, Dock-Nascimento DB, Correia MI, Campos AC, Portari-Filho PE, Oliveira SS (2014). Actual preoperative fasting time in Brazilian hospitals the BIGFAST multicenter study. Ther Clin Risk Manag.

[5] Aguilar-Nascimento JE, Marra JG, Slhessarenko N, Fontes CJ (2007). Efficacy of National Nosocomial Infection Surveillance score, acute-phase proteins, and interleukin-6 for predicting postoperative infections following major gastrointestinal surgery. Sao Paulo Med J.

[6] Aguilar-Nascimento JE, Bicudo-Salomão A, Caporossi C, Silva RM, Cardoso EA, Santos TP, Diniz BN, Hartmann AA (2009). Abordagem multimodal em cirurgia colorretal sem preparo mecânico de cólon. Rev Col Bras Cir.

[7] Aguilar-Nascimento JE, Dock-Nascimento DB, Faria MSM, Maria EV, Yonamine F, Silva MR, Adler T (2008). Ingestão pré-operatória de carboidratos diminui a ocorrência de sintomas gastrointestinais pós-operatórios em pacientes submetidos à colecistectomia. ABCD. Arquivos Brasileiros de Cirurgia Digestiva.

[8] Ahmed J, Lim M, Khan S, McNaught C, Macfie J (2010). Predictors of length of stay in patients having elective colorectal surgery within an enhanced recovery protocol. Int J Surg.

[9] Bicudo-Salomão A, Meireles MB, Caporossi C, Crotti PLR, Aguilar-Nascimento JE (2011). Impact of the acerto project in the postoperative morbi-mortality in a university hospital Rev Col Bras. Cir.

[10] Culver DH, Horan TC, Gaynes RP (1991). Surgical wound infection rates by wound class, operative procedure, and patient risk index National Nosocomial Infections Surveillance System. Am J Med.

[11] de-Aguilar-Nascimento JE, Salomão AB, Waitzberg DL, Dock-Nascimento DB, Correa MITD, Campos ACL, Corsi PR, Portari PE, Caporossi C (2017). Diretriz ACERTO de intervenções nutricionais no perioperatório em cirurgia geral eletiva. Rev Col Bras Cir.

[12] Fernandez OOA, Pereira JA, Campos FG, Araya CM, Marinho GE, Novo RS, Oliveira TS, Franceschi YT, Martinez CAR (2018). Evaluation of enemas containing sucralfate in tissue content of MUC-2 protein in experimental model of diversion colitis. Arq Bras Cir Dig.

[13] Garner JS, Jarvis WR, Emori TG, Horan TC, Hughes JM (1988). CDC definitions for nosocomial infections, 1988. Am J Infect Control.

[14] Gatt M, Anderson AD, Reddy BS, Hayward-Sampson P, Tring IC, MacFie J (2005). Randomized clinical trial of multimodal optimization of surgical care in patients undergoing major colonic resection. Br J Surg.

[15] Gustafsson UO, Hausel J, Thorell A, Ljungqvist O, Soop M, Nygren J, Enhanced Recovery After Surgery Study Group (2011). Adherence to the enhanced recovery after surgery protocol and outcomes after colorectal cancer surgery. Arch Surg.

[16] Khoo CK, Vickery CJ, Forsyth N, Vinall NS, Eyre-Brook IA (2007). A prospective randomized controlled trial of multimodal perioperative management protocol in patients undergoing elective colorectal resection for cancer. Ann Surg.

[17] King PM, Blazeby JM, Ewings P, Franks PJ, Longman RJ, Kendrick AH, Kipling RM, Kennedy RH (2006). Randomized clinical trial comparing laparoscopic and open surgery for colorectal cancer within an enhanced recovery programme. Br J Surg.

[18] Ljungqvist O (2014). ERAS--Enhanced Recovery After Surgery: Moving Evidence-Based Perioperative Care to Practice. JPEN J Parenter Enteral Nutr.

[19] Lv L, Shao YF, Zhou YB (2012). The enhanced recovery after surgery (ERAS) pathway for patients undergoing colorectal surgery: an update of meta-analysis of randomized controlled trials. Int J Colorectal Dis.

[20] Muller S, Zalunardo MP, Hubner M, Clavien PA, Demartines N (2009). A fast-track program reduces complications and length of hospital stay after open colonic surgery. Gastroenterology.

[21] Ni X, Jia D, Chen Y, Wang L, Suo J (2019). Is the Enhanced Recovery After Surgery (ERAS) Program Effective and Safe in Laparoscopic Colorectal Cancer Surgery? A Meta-Analysis of Randomized Controlled Trials. J Gastrointest Surg.

[22] Ozhathil DK, Li Y, Smith JK, Witkowski E, Coyne ER, Alavi K, Tseng JF, Shah SA (2011). Colectomy performance improvement within NSQIP 2005-2008. J Surg Res.

[23] Silva CED, Repka JCD, Souza CJF, Matias JEF (2018). Effects of renal dysfunction on healing of colonic anastomosis experimental study in wistar rats. Arq Bras Cir Dig.

[24] Spanjersberg WR, Reurings J, Keus F, van Laarhoven CJ (2011). Fast track surgery versus conventional recovery strategies for colorectal surgery. Cochrane Database Syst Rev.

[25] Teixeira UF, Fontes PRO, Conceição CWN, Farias CAT, Fernandes D, Ewald IP i (2019). Implementation of enhanced recovery after colorectal surgery (eras) protocol initial results of the first brazilian experience.. ABCD Arq. Bras. Cir. Dig.

[26] Teixeira UF, Goldoni MB, Waechter FL, Sampaio JA, Mendes FF, Fontes PRO (2019). Enhanced recovery (ERAS) after liver surgery comparative study in a brazilian terciary center. ABCD Arq. Bras. Cir. Dig.

[27] Tekkis PP, Prytherch DR, Kocher HM, Senapati A, Poloniecki JD, Stamatakis JD, Windsor AC (2004). Development of a dedicated risk-adjustment scoring system for colorectal surgery (colorectal POSSUM). Br J Surg.

[28] Turina M, Remzi FH, Dietz DW, Kiran RP, Seyidova-Khoshknabi D, Hammel JP, Vogel JD (2013). Quantification of risk for early unplanned readmission after rectal resection a single-center study. J Am Coll Surg.

[29] Varadhan KK, Neal KR, Dejong CH, Fearon KC, Ljungqvist O, Lobo DN (2010). The enhanced recovery after surgery (ERAS) pathway for patients undergoing major elective open colorectal surgery a meta-analysis of randomized controlled trials. ClinNutr.

[30] Waitzberg DL, Caiaffa WT, Correia MI (2001). Hospital malnutrition: the Brazilian national survey (IBRANUTRI): a study of 4000 patients. Nutrition.

[31] Wind J, Polle SW, Fung Kon Jin PH (2006). Systematic review of enhanced recovery programmes in colonic surgery. Br J Surg.

[32] Zhuang CL, Ye XZ, Zhang XD, Chen BC, Yu Z (2013). Enhanced recovery after surgery programs versus traditional care for colorectal surgery a meta-analysis of randomized controlled trials. Dis Colon Rectum.

